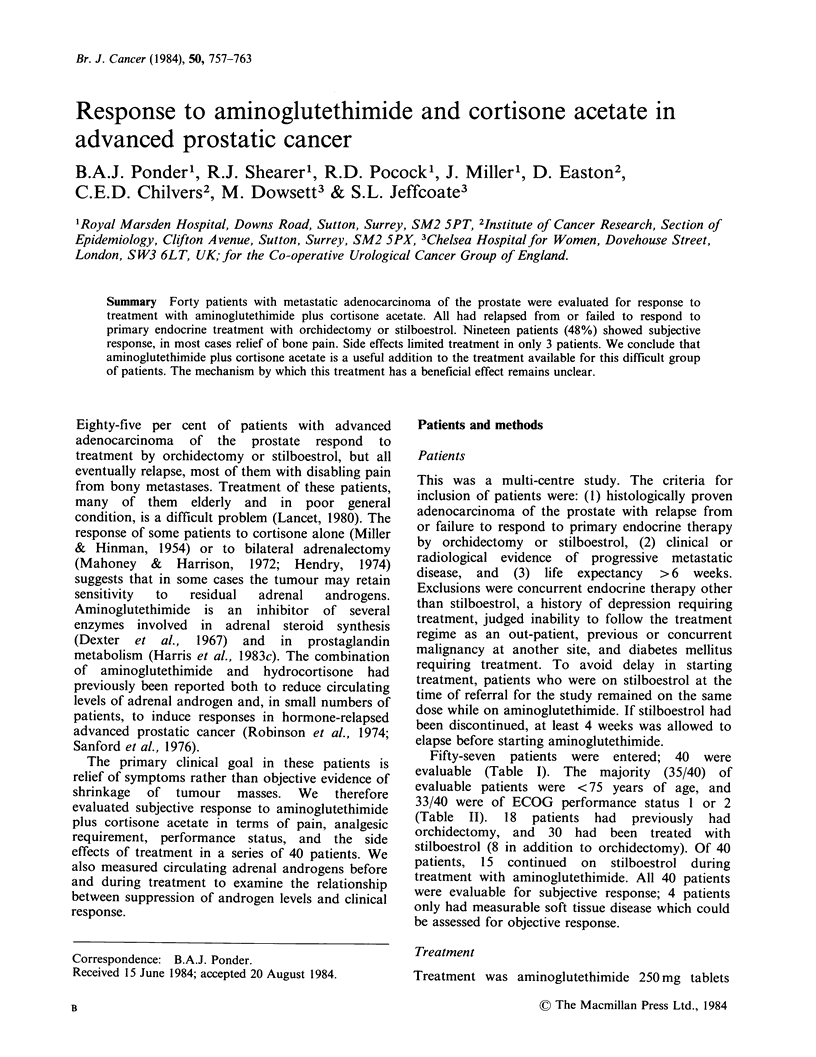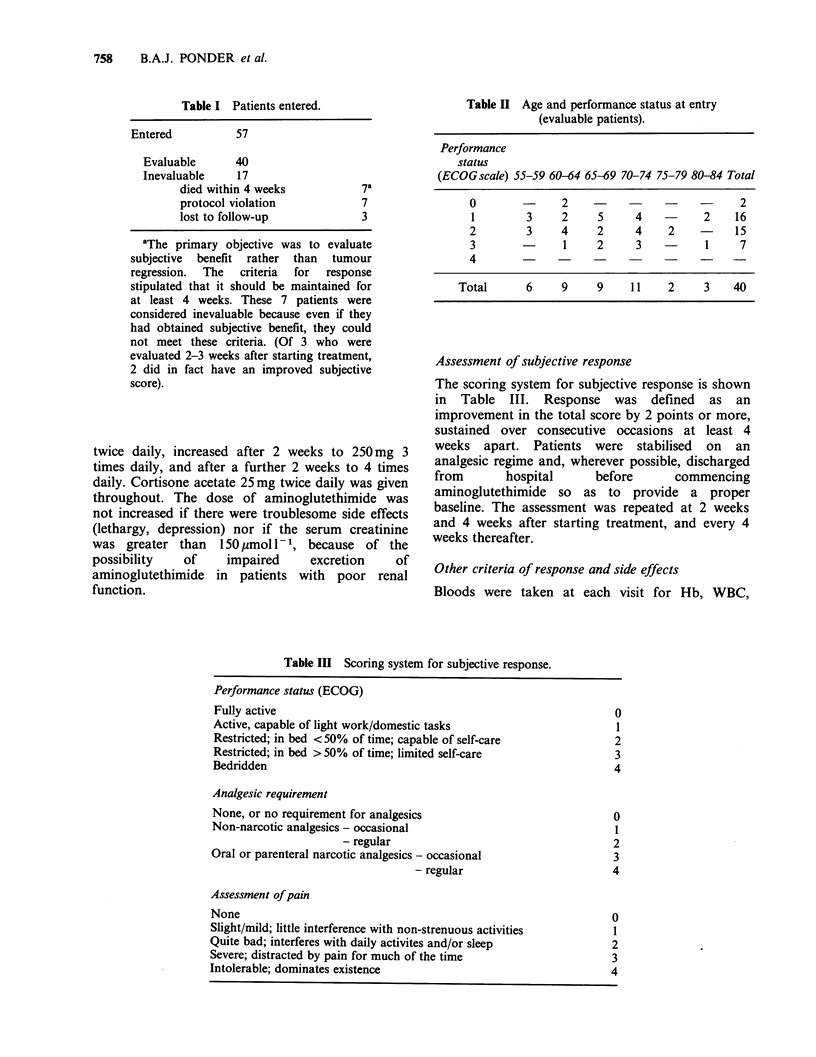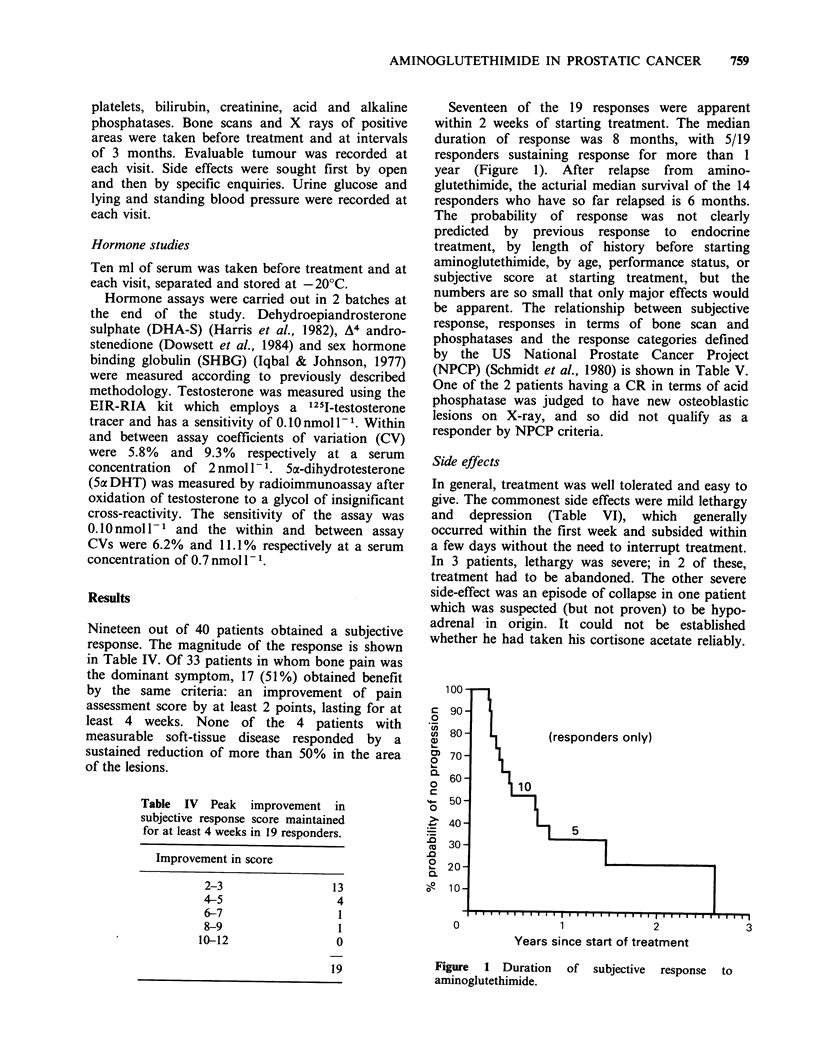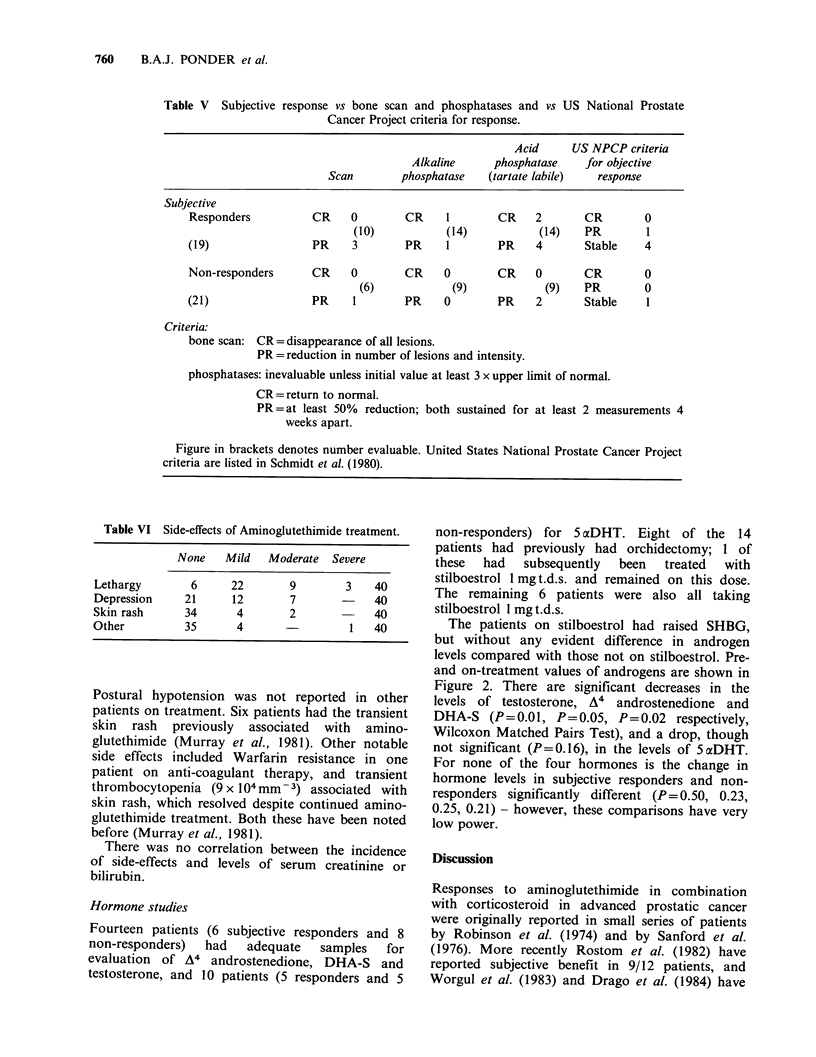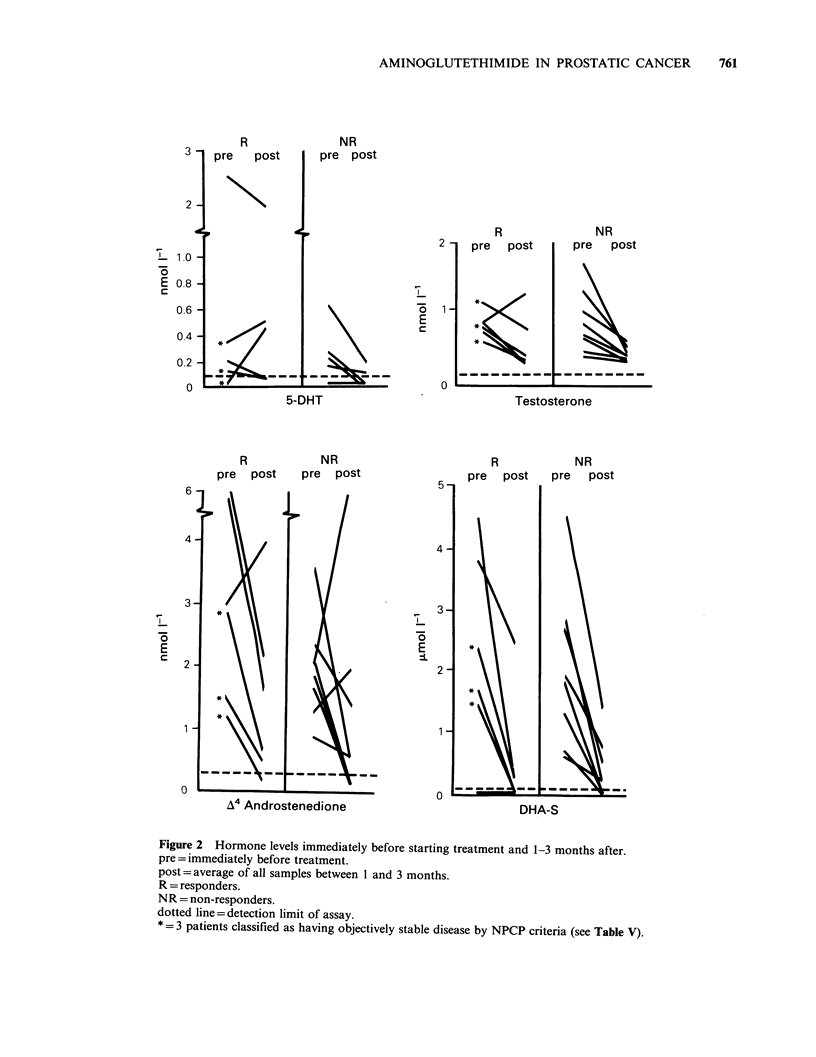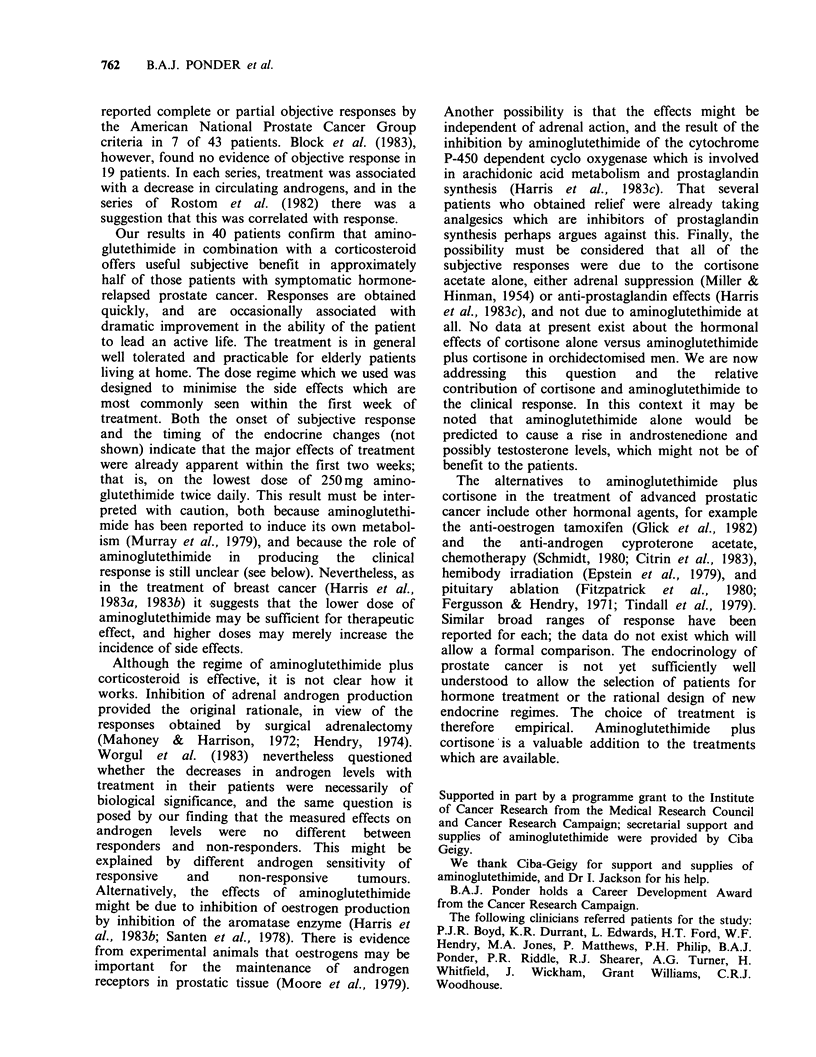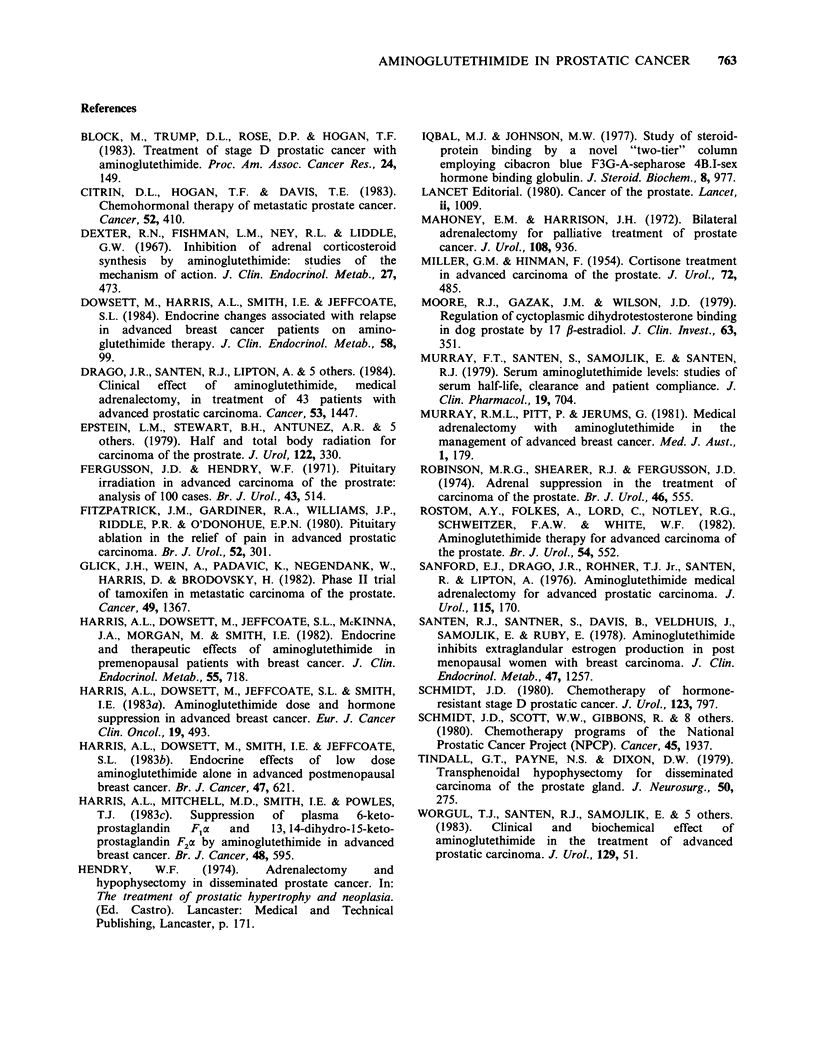# Response to aminoglutethimide and cortisone acetate in advanced prostatic cancer.

**DOI:** 10.1038/bjc.1984.253

**Published:** 1984-12

**Authors:** B. A. Ponder, R. J. Shearer, R. D. Pocock, J. Miller, D. Easton, C. E. Chilvers, M. Dowsett, S. L. Jeffcoate

## Abstract

Forty patients with metastatic adenocarcinoma of the prostate were evaluated for response to treatment with aminoglutethimide plus cortisone acetate. All had relapsed from or failed to respond to primary endocrine treatment with orchidectomy or stilboestrol. Nineteen patients (48%) showed subjective response, in most cases relief of bone pain. Side effects limited treatment in only 3 patients. We conclude that aminoglutethimide plus cortisone acetate is a useful addition to the treatment available for this difficult group of patients. The mechanism by which this treatment has a beneficial effect remains unclear.


*Br. J. Cancer* (1984), **50**, 757–763

### Response to aminoglutethimide and cortisone acetate in advanced prostatic cancer

B.A.J. Ponder[1], R.J. Shearer[1], R.D. Pocock[1], J. Miller[1], D. Easton[2], C.E.D. Chilvers[2], M. Dowsett[3] & S.L. Jeffcoate[3]

[1]*Royal Marsden Hospital, Downs Road, Sutton, Surrey, SM2 5PT,* [2]*Institute of Cancer Research, Section of Epidemiology, Clifton Avenue, Sutton, Surrey, SM2 5PX,* [3]*Chelsea Hospital for Women, Dovehouse Street, London, SW3 6LT, UK; for the Co-operative Urological Cancer Group of England.*

**Summary** Forty patients with metastatic adenocarcinoma of the prostate were evaluated for response to treatment with aminoglutethimide plus cortisone acetate. All had relapsed from or failed to respond to primary endocrine treatment with orchidectomy or stilboestrol. Nineteen patients (48%) showed subjective response, in most cases relief of bone pain. Side effects limited treatment in only 3 patients. We conclude that aminoglutethimide plus cortisone acetate is a useful addition to the treatment available for this difficult group of patients. The mechanism by which this treatment has a beneficial effect remains unclear.

Eighty-five per cent of patients with advanced adenocarcinoma of the prostate respond to treatment by orchidectomy or stilboestrol, but all eventually relapse, most of them with disabling pain from bony metastases. Treatment of these patients, many of them elderly and in poor general condition, is a difficult problem (Lancet, 1980). The response of some patients to cortisone alone (Miller & Hinman, 1954) or to bilateral adrenalectomy (Mahoney & Harrison, 1972; Hendry, 1974) suggests that in some cases the tumour may retain sensitivity to residual adrenal androgens. Aminoglutethimide is an inhibitor of several enzymes involved in adrenal steroid synthesis (Dexter *et al.*, 1967) and in prostaglandin metabolism (Harris *et al.*, 1983*c*). The combination of aminoglutethimide and hydrocortisone had previously been reported both to reduce circulating levels of adrenal androgen and, in small numbers of patients, to induce responses in hormone-relapsed advanced prostatic cancer (Robinson *et al.*, 1974; Sanford *et al.*, 1976).

The primary clinical goal in these patients is relief of symptoms rather than objective evidence of shrinkage of tumour masses. We therefore evaluated subjective response to aminoglutethimide plus cortisone acetate in terms of pain, analgesic requirement, performance status, and the side effects of treatment in a series of 40 patients. We also measured circulating adrenal androgens before and during treatment to examine the relationship between suppression of androgen levels and clinical response.

Correspondence: B.A.J. Ponder.
Received 15 June 1984; accepted 20 August 1984.

### Patients and methods

### *Patients*

This was a multi-centre study. The criteria for inclusion of patients were: (1) histologically proven adenocarcinoma of the prostate with relapse from or failure to respond to primary endocrine therapy by orchidectomy or stilboestrol, (2) clinical or radiological evidence of progressive metastatic disease, and (3) life expectancy >6 weeks. Exclusions were concurrent endocrine therapy other than stilboestrol, a history of depression requiring treatment, judged inability to follow the treatment regime as an out-patient, previous or concurrent malignancy at another site, and diabetes mellitus requiring treatment. To avoid delay in starting treatment, patients who were on stilboestrol at the time of referral for the study remained on the same dose while on aminoglutethimide. If stilboestrol had been discontinued, at least 4 weeks was allowed to elapse before starting aminoglutethimide.

Fifty-seven patients were entered; 40 were evaluable (Table I). The majority (35/40) of evaluable patients were <75 years of age, and 33/40 were of ECOG performance status 1 or 2 (Table II). 18 patients had previously had orchidectomy, and 30 had been treated with stilboestrol (8 in addition to orchidectomy). Of 40 patients, 15 continued on stilboestrol during treatment with aminoglutethimide. All 40 patients were evaluable for subjective response; 4 patients only had measurable soft tissue disease which could be assessed for objective response.

### *Treatment*

Treatment was aminoglutethimide 250 mg tablets

© The Macmillan Press Ltd., 1984

**Table I** Patients entered.

| | | |
|---|---|---|
| Entered | 57 | |
| Evaluable | 40 | |
| Inevaluable | 17 | |
| died within 4 weeks | | 7[a] |
| protocol violation | | 7 |
| lost to follow-up | | 3 |

[a]The primary objective was to evaluate subjective benefit rather than tumour regression. The criteria for response stipulated that it should be maintained for at least 4 weeks. These 7 patients were considered inevaluable because even if they had obtained subjective benefit, they could not meet these criteria. (Of 3 who were evaluated 2–3 weeks after starting treatment, 2 did in fact have an improved subjective score).

**Table II** Age and performance status at entry (evaluable patients).

| *Performance status (ECOG scale)* | *55–59* | *60–64* | *65–69* | *70–74* | *75–79* | *80–84* | *Total* |
|---|---|---|---|---|---|---|---|
| 0 | — | 2 | — | — | — | — | 2 |
| 1 | 3 | 2 | 5 | 4 | — | 2 | 16 |
| 2 | 3 | 4 | 2 | 4 | 2 | — | 15 |
| 3 | — | 1 | 2 | 3 | — | 1 | 7 |
| 4 | — | — | — | — | — | — | — |
| Total | 6 | 9 | 9 | 11 | 2 | 3 | 40 |

twice daily, increased after 2 weeks to 250 mg 3 times daily, and after a further 2 weeks to 4 times daily. Cortisone acetate 25 mg twice daily was given throughout. The dose of aminoglutethimide was not increased if there were troublesome side effects (lethargy, depression) nor if the serum creatinine was greater than 150 $\mu mol\,l^{-1}$, because of the possibility of impaired excretion of aminoglutethimide in patients with poor renal function.

### *Assessment of subjective response*

The scoring system for subjective response is shown in Table III. Response was defined as an improvement in the total score by 2 points or more, sustained over consecutive occasions at least 4 weeks apart. Patients were stabilised on an analgesic regime and, wherever possible, discharged from hospital before commencing aminoglutethimide so as to provide a proper baseline. The assessment was repeated at 2 weeks and 4 weeks after starting treatment, and every 4 weeks thereafter.

### *Other criteria of response and side effects*

Bloods were taken at each visit for Hb, WBC,

**Table III** Scoring system for subjective response.

| | |
|---|---|
| *Performance status* (ECOG) | |
| Fully active | 0 |
| Active, capable of light work/domestic tasks | 1 |
| Restricted; in bed <50% of time; capable of self-care | 2 |
| Restricted; in bed >50% of time; limited self-care | 3 |
| Bedridden | 4 |
| *Analgesic requirement* | |
| None, or no requirement for analgesics | 0 |
| Non-narcotic analgesics – occasional | 1 |
| – regular | 2 |
| Oral or parenteral narcotic analgesics – occasional | 3 |
| – regular | 4 |
| *Assessment of pain* | |
| None | 0 |
| Slight/mild; little interference with non-strenuous activities | 1 |
| Quite bad; interferes with daily activites and/or sleep | 2 |
| Severe; distracted by pain for much of the time | 3 |
| Intolerable; dominates existence | 4 |

platelets, bilirubin, creatinine, acid and alkaline phosphatases. Bone scans and X rays of positive areas were taken before treatment and at intervals of 3 months. Evaluable tumour was recorded at each visit. Side effects were sought first by open and then by specific enquiries. Urine glucose and lying and standing blood pressure were recorded at each visit.

*Hormone studies*

Ten ml of serum was taken before treatment and at each visit, separated and stored at −20°C.

Hormone assays were carried out in 2 batches at the end of the study. Dehydroepiandrosterone sulphate (DHA-S) (Harris *et al.,* 1982), $\Delta^4$ androstenedione (Dowsett *et al.,* 1984) and sex hormone binding globulin (SHBG) (Iqbal & Johnson, 1977) were measured according to previously described methodology. Testosterone was measured using the EIR-RIA kit which employs a $^{125}$I-testosterone tracer and has a sensitivity of 0.10 nmol l$^{-1}$. Within and between assay coefficients of variation (CV) were 5.8% and 9.3% respectively at a serum concentration of 2 nmol l$^{-1}$. 5α-dihydrotestosterone (5α DHT) was measured by radioimmunoassay after oxidation of testosterone to a glycol of insignificant cross-reactivity. The sensitivity of the assay was 0.10 nmol l$^{-1}$ and the within and between assay CVs were 6.2% and 11.1% respectively at a serum concentration of 0.7 nmol l$^{-1}$.

### Results

Nineteen out of 40 patients obtained a subjective response. The magnitude of the response is shown in Table IV. Of 33 patients in whom bone pain was the dominant symptom, 17 (51%) obtained benefit by the same criteria: an improvement of pain assessment score by at least 2 points, lasting for at least 4 weeks. None of the 4 patients with measurable soft-tissue disease responded by a sustained reduction of more than 50% in the area of the lesions.

**Table IV** Peak improvement in subjective response score maintained for at least 4 weeks in 19 responders.

| Improvement in score | |
|---|---|
| 2–3 | 13 |
| 4–5 | 4 |
| 6–7 | 1 |
| 8–9 | 1 |
| 10–12 | 0 |
| | 19 |

Seventeen of the 19 responses were apparent within 2 weeks of starting treatment. The median duration of response was 8 months, with 5/19 responders sustaining response for more than 1 year (Figure 1). After relapse from aminoglutethimide, the acturial median survival of the 14 responders who have so far relapsed is 6 months. The probability of response was not clearly predicted by previous response to endocrine treatment, by length of history before starting aminoglutethimide, by age, performance status, or subjective score at starting treatment, but the numbers are so small that only major effects would be apparent. The relationship between subjective response, responses in terms of bone scan and phosphatases and the response categories defined by the US National Prostate Cancer Project (NPCP) (Schmidt *et al.,* 1980) is shown in Table V. One of the 2 patients having a CR in terms of acid phosphatase was judged to have new osteoblastic lesions on X-ray, and so did not qualify as a responder by NPCP criteria.

*Side effects*

In general, treatment was well tolerated and easy to give. The commonest side effects were mild lethargy and depression (Table VI), which generally occurred within the first week and subsided within a few days without the need to interrupt treatment. In 3 patients, lethargy was severe; in 2 of these, treatment had to be abandoned. The other severe side-effect was an episode of collapse in one patient which was suspected (but not proven) to be hypoadrenal in origin. It could not be established whether he had taken his cortisone acetate reliably.

**Figure 1** Duration of subjective response to aminoglutethimide.

**Table V** Subjective response *vs* bone scan and phosphatases and *vs* US National Prostate Cancer Project criteria for response.

| | Scan | | Alkaline phosphatase | | Acid phosphatase (tartate labile) | | US NPCP criteria for objective response | |
|---|---|---|---|---|---|---|---|---|
| *Subjective* | | | | | | | | |
| Responders | CR | 0 | CR | 1 | CR | 2 | CR | 0 |
| | | (10) | | (14) | | (14) | PR | 1 |
| (19) | PR | 3 | PR | 1 | PR | 4 | Stable | 4 |
| Non-responders | CR | 0 | CR | 0 | CR | 0 | CR | 0 |
| | | (6) | | (9) | | (9) | PR | 0 |
| (21) | PR | 1 | PR | 0 | PR | 2 | Stable | 1 |

*Criteria:*

bone scan: CR = disappearance of all lesions.
PR = reduction in number of lesions and intensity.

phosphatases: inevaluable unless initial value at least 3 × upper limit of normal.

CR = return to normal.
PR = at least 50% reduction; both sustained for at least 2 measurements 4 weeks apart.

Figure in brackets denotes number evaluable. United States National Prostate Cancer Project criteria are listed in Schmidt *et al.* (1980).

**Table VI** Side-effects of Aminoglutethimide treatment.

| | *None* | *Mild* | *Moderate* | *Severe* | |
|---|---|---|---|---|---|
| Lethargy | 6 | 22 | 9 | 3 | 40 |
| Depression | 21 | 12 | 7 | — | 40 |
| Skin rash | 34 | 4 | 2 | — | 40 |
| Other | 35 | 4 | — | 1 | 40 |

Postural hypotension was not reported in other patients on treatment. Six patients had the transient skin rash previously associated with aminoglutethimide (Murray *et al.*, 1981). Other notable side effects included Warfarin resistance in one patient on anti-coagulant therapy, and transient thrombocytopenia ($9 \times 10^4 mm^{-3}$) associated with skin rash, which resolved despite continued aminoglutethimide treatment. Both these have been noted before (Murray *et al.*, 1981).

There was no correlation between the incidence of side-effects and levels of serum creatinine or bilirubin.

*Hormone studies*

Fourteen patients (6 subjective responders and 8 non-responders) had adequate samples for evaluation of $\Delta^4$ androstenedione, DHA-S and testosterone, and 10 patients (5 responders and 5 non-responders) for 5αDHT. Eight of the 14 patients had previously had orchidectomy; 1 of these had subsequently been treated with stilboestrol 1 mg t.d.s. and remained on this dose. The remaining 6 patients were also all taking stilboestrol 1 mg t.d.s.

The patients on stilboestrol had raised SHBG, but without any evident difference in androgen levels compared with those not on stilboestrol. Pre- and on-treatment values of androgens are shown in Figure 2. There are significant decreases in the levels of testosterone, $\Delta^4$ androstenedione and DHA-S ($P=0.01$, $P=0.05$, $P=0.02$ respectively, Wilcoxon Matched Pairs Test), and a drop, though not significant ($P=0.16$), in the levels of 5αDHT. For none of the four hormones is the change in hormone levels in subjective responders and non-responders significantly different ($P=0.50$, 0.23, 0.25, 0.21) – however, these comparisons have very low power.

### Discussion

Responses to aminoglutethimide in combination with corticosteroid in advanced prostatic cancer were originally reported in small series of patients by Robinson *et al.* (1974) and by Sanford *et al.* (1976). More recently Rostom *et al.* (1982) have reported subjective benefit in 9/12 patients, and Worgul *et al.* (1983) and Drago *et al.* (1984) have

**Figure 2** Hormone levels immediately before starting treatment and 1–3 months after.
pre = immediately before treatment.
post = average of all samples between 1 and 3 months.
R = responders.
NR = non-responders.
dotted line = detection limit of assay.
* = 3 patients classified as having objectively stable disease by NPCP criteria (see **Table V**).

reported complete or partial objective responses by the American National Prostate Cancer Group criteria in 7 of 43 patients. Block *et al.* (1983), however, found no evidence of objective response in 19 patients. In each series, treatment was associated with a decrease in circulating androgens, and in the series of Rostom *et al.* (1982) there was a suggestion that this was correlated with response.

Our results in 40 patients confirm that aminoglutethimide in combination with a corticosteroid offers useful subjective benefit in approximately half of those patients with symptomatic hormone-relapsed prostate cancer. Responses are obtained quickly, and are occasionally associated with dramatic improvement in the ability of the patient to lead an active life. The treatment is in general well tolerated and practicable for elderly patients living at home. The dose regime which we used was designed to minimise the side effects which are most commonly seen within the first week of treatment. Both the onset of subjective response and the timing of the endocrine changes (not shown) indicate that the major effects of treatment were already apparent within the first two weeks; that is, on the lowest dose of 250 mg aminoglutethimide twice daily. This result must be interpreted with caution, both because aminoglutethimide has been reported to induce its own metabolism (Murray *et al.*, 1979), and because the role of aminoglutethimide in producing the clinical response is still unclear (see below). Nevertheless, as in the treatment of breast cancer (Harris *et al.*, 1983*a*, 1983*b*) it suggests that the lower dose of aminoglutethimide may be sufficient for therapeutic effect, and higher doses may merely increase the incidence of side effects.

Although the regime of aminoglutethimide plus corticosteroid is effective, it is not clear how it works. Inhibition of adrenal androgen production provided the original rationale, in view of the responses obtained by surgical adrenalectomy (Mahoney & Harrison, 1972; Hendry, 1974). Worgul *et al.* (1983) nevertheless questioned whether the decreases in androgen levels with treatment in their patients were necessarily of biological significance, and the same question is posed by our finding that the measured effects on androgen levels were no different between responders and non-responders. This might be explained by different androgen sensitivity of responsive and non-responsive tumours. Alternatively, the effects of aminoglutethimide might be due to inhibition of oestrogen production by inhibition of the aromatase enzyme (Harris *et al.*, 1983*b*; Santen *et al.*, 1978). There is evidence from experimental animals that oestrogens may be important for the maintenance of androgen receptors in prostatic tissue (Moore *et al.*, 1979). Another possibility is that the effects might be independent of adrenal action, and the result of the inhibition by aminoglutethimide of the cytochrome P-450 dependent cyclo oxygenase which is involved in arachidonic acid metabolism and prostaglandin synthesis (Harris *et al.*, 1983*c*). That several patients who obtained relief were already taking analgesics which are inhibitors of prostaglandin synthesis perhaps argues against this. Finally, the possibility must be considered that all of the subjective responses were due to the cortisone acetate alone, either adrenal suppression (Miller & Hinman, 1954) or anti-prostaglandin effects (Harris *et al.*, 1983*c*), and not due to aminoglutethimide at all. No data at present exist about the hormonal effects of cortisone alone versus aminoglutethimide plus cortisone in orchidectomised men. We are now addressing this question and the relative contribution of cortisone and aminoglutethimide to the clinical response. In this context it may be noted that aminoglutethimide alone would be predicted to cause a rise in androstenedione and possibly testosterone levels, which might not be of benefit to the patients.

The alternatives to aminoglutethimide plus cortisone in the treatment of advanced prostatic cancer include other hormonal agents, for example the anti-oestrogen tamoxifen (Glick *et al.*, 1982) and the anti-androgen cyproterone acetate, chemotherapy (Schmidt, 1980; Citrin *et al.*, 1983), hemibody irradiation (Epstein *et al.*, 1979), and pituitary ablation (Fitzpatrick *et al.*, 1980; Fergusson & Hendry, 1971; Tindall *et al.*, 1979). Similar broad ranges of response have been reported for each; the data do not exist which will allow a formal comparison. The endocrinology of prostate cancer is not yet sufficiently well understood to allow the selection of patients for hormone treatment or the rational design of new endocrine regimes. The choice of treatment is therefore empirical. Aminoglutethimide plus cortisone is a valuable addition to the treatments which are available.

Supported in part by a programme grant to the Institute of Cancer Research from the Medical Research Council and Cancer Research Campaign; secretarial support and supplies of aminoglutethimide were provided by Ciba Geigy.

We thank Ciba-Geigy for support and supplies of aminoglutethimide, and Dr I. Jackson for his help.

B.A.J. Ponder holds a Career Development Award from the Cancer Research Campaign.

The following clinicians referred patients for the study: P.J.R. Boyd, K.R. Durrant, L. Edwards, H.T. Ford, W.F. Hendry, M.A. Jones, P. Matthews, P.H. Philip, B.A.J. Ponder, P.R. Riddle, R.J. Shearer, A.G. Turner, H. Whitfield, J. Wickham, Grant Williams, C.R.J. Woodhouse.

**References**

BLOCK, M., TRUMP, D.L., ROSE, D.P. & HOGAN, T.F. (1983). Treatment of stage D prostatic cancer with aminoglutethimide. *Proc. Am. Assoc. Cancer Res.*, **24**, 149.

CITRIN, D.L., HOGAN, T.F. & DAVIS, T.E. (1983). Chemohormonal therapy of metastatic prostate cancer. *Cancer*, **52**, 410.

DEXTER, R.N., FISHMAN, L.M., NEY, R.L. & LIDDLE, G.W. (1967). Inhibition of adrenal corticosteroid synthesis by aminoglutethimide: studies of the mechanism of action. *J. Clin. Endocrinol. Metab.*, **27**, 473.

DOWSETT, M., HARRIS, A.L., SMITH, I.E. & JEFFCOATE, S.L. (1984). Endocrine changes associated with relapse in advanced breast cancer patients on aminoglutethimide therapy. *J. Clin. Endocrinol. Metab.*, **58**, 99.

DRAGO, J.R., SANTEN, R.J., LIPTON, A. & 5 others. (1984). Clinical effect of aminoglutethimide, medical adrenalectomy, in treatment of 43 patients with advanced prostatic carcinoma. *Cancer*, **53**, 1447.

EPSTEIN, L.M., STEWART, B.H., ANTUNEZ, A.R. & 5 others. (1979). Half and total body radiation for carcinoma of the prostrate. *J. Urol*, **122**, 330.

FERGUSSON, J.D. & HENDRY, W.F. (1971). Pituitary irradiation in advanced carcinoma of the prostrate: analysis of 100 cases. *Br. J. Urol.*, **43**, 514.

FITZPATRICK, J.M., GARDINER, R.A., WILLIAMS, J.P., RIDDLE, P.R. & O'DONOHUE, E.P.N. (1980). Pituitary ablation in the relief of pain in advanced prostatic carcinoma. *Br. J. Urol.*, **52**, 301.

GLICK, J.H., WEIN, A., PADAVIC, K., NEGENDANK, W., HARRIS, D. & BRODOVSKY, H. (1982). Phase II trial of tamoxifen in metastatic carcinoma of the prostate. *Cancer*, **49**, 1367.

HARRIS, A.L., DOWSETT, M., JEFFCOATE, S.L., McKINNA, J.A., MORGAN, M. & SMITH, I.E. (1982). Endocrine and therapeutic effects of aminoglutethimide in premenopausal patients with breast cancer. *J. Clin. Endocrinol. Metab.*, **55**, 718.

HARRIS, A.L., DOWSETT, M., JEFFCOATE, S.L. & SMITH, I.E. (1983*a*). Aminoglutethimide dose and hormone suppression in advanced breast cancer. *Eur. J. Cancer Clin. Oncol.*, **19**, 493.

HARRIS, A.L., DOWSETT, M., SMITH, I.E. & JEFFCOATE, S.L. (1983*b*). Endocrine effects of low dose aminoglutethimide alone in advanced postmenopausal breast cancer. *Br. J. Cancer*, **47**, 621.

HARRIS, A.L., MITCHELL, M.D., SMITH, I.E. & POWLES, T.J. (1983*c*). Suppression of plasma 6-keto-prostaglandin $F_1\alpha$ and 13,14-dihydro-15-keto-prostaglandin $F_2\alpha$ by aminoglutethimide in advanced breast cancer. *Br. J. Cancer*, **48**, 595.

HENDRY, W.F. (1974). Adrenalectomy and hypophysectomy in disseminated prostate cancer. In: *The treatment of prostatic hypertrophy and neoplasia.* (Ed. Castro). Lancaster: Medical and Technical Publishing, Lancaster, p. 171.

IQBAL, M.J. & JOHNSON, M.W. (1977). Study of steroid-protein binding by a novel "two-tier" column employing cibacron blue F3G-A-sepharose 4B.I-sex hormone binding globulin. *J. Steroid. Biochem.*, **8**, 977.

LANCET Editorial. (1980). Cancer of the prostate. *Lancet*, **ii**, 1009.

MAHONEY, E.M. & HARRISON, J.H. (1972). Bilateral adrenalectomy for palliative treatment of prostate cancer. *J. Urol.*, **108**, 936.

MILLER, G.M. & HINMAN, F. (1954). Cortisone treatment in advanced carcinoma of the prostate. *J. Urol.*, **72**, 485.

MOORE, R.J., GAZAK, J.M. & WILSON, J.D. (1979). Regulation of cyctoplasmic dihydrotestosterone binding in dog prostate by 17 $\beta$-estradiol. *J. Clin. Invest.*, **63**, 351.

MURRAY, F.T., SANTEN, S., SAMOJLIK, E. & SANTEN, R.J. (1979). Serum aminoglutethimide levels: studies of serum half-life, clearance and patient compliance. *J. Clin. Pharmacol.*, **19**, 704.

MURRAY, R.M.L., PITT, P. & JERUMS, G. (1981). Medical adrenalectomy with aminoglutethimide in the management of advanced breast cancer. *Med. J. Aust.*, **1**, 179.

ROBINSON, M.R.G., SHEARER, R.J. & FERGUSSON, J.D. (1974). Adrenal suppression in the treatment of carcinoma of the prostate. *Br. J. Urol.*, **46**, 555.

ROSTOM, A.Y., FOLKES, A., LORD, C., NOTLEY, R.G., SCHWEITZER, F.A.W. & WHITE, W.F. (1982). Aminoglutethimide therapy for advanced carcinoma of the prostate. *Br. J. Urol.*, **54**, 552.

SANFORD, E.J., DRAGO, J.R., ROHNER, T.J. Jr., SANTEN, R. & LIPTON, A. (1976). Aminoglutethimide medical adrenalectomy for advanced prostatic carcinoma. *J. Urol.*, **115**, 170.

SANTEN, R.J., SANTNER, S., DAVIS, B., VELDHUIS, J., SAMOJLIK, E. & RUBY, E. (1978). Aminoglutethimide inhibits extraglandular estrogen production in post menopausal women with breast carcinoma. *J. Clin. Endocrinol. Metab.*, **47**, 1257.

SCHMIDT, J.D. (1980). Chemotherapy of hormone-resistant stage D prostatic cancer. *J. Urol.*, **123**, 797.

SCHMIDT, J.D., SCOTT, W.W., GIBBONS, R. & 8 others. (1980). Chemotherapy programs of the National Prostatic Cancer Project (NPCP). *Cancer*, **45**, 1937.

TINDALL, G.T., PAYNE, N.S. & DIXON, D.W. (1979). Transphenoidal hypophysectomy for disseminated carcinoma of the prostate gland. *J. Neurosurg.*, **50**, 275.

WORGUL, T.J., SANTEN, R.J., SAMOJLIK, E. & 5 others. (1983). Clinical and biochemical effect of aminoglutethimide in the treatment of advanced prostatic carcinoma. *J. Urol.*, **129**, 51.